# Autism-associated variants of neuroligin 4X impair synaptogenic activity by various molecular mechanisms

**DOI:** 10.1186/s13229-020-00373-y

**Published:** 2020-09-01

**Authors:** Takafumi Yumoto, Misaki Kimura, Ryota Nagatomo, Tsukika Sato, Shun Utsunomiya, Natsue Aoki, Motoji Kitaura, Koji Takahashi, Hiroshi Takemoto, Hirotaka Watanabe, Hideyuki Okano, Fumiaki Yoshida, Yosuke Nao, Taisuke Tomita

**Affiliations:** 1 Laboratory of Neuropathology and Neuroscience, Graduate School of Pharmaceutical Sciences, The University of Tokyo, 7-3-1 Hongo, Bunkyo-ku, Tokyo, 113-0033 Japan; 2 Department of Physiology, Keio University School of Medicine, Tokyo, Japan; 3 Neuroscience 2, Laboratory for Drug Discovery and Disease Research, Shionogi, Osaka, Japan; 4 Business-Academia Collaborative Laboratory (Shionogi), Graduate School of Pharmaceutical Science, The University of Tokyo, Tokyo, Japan; 5 Research Administration SPRC, R&D General Administration Unit, General Administration Division, Shionogi Administration Service, Osaka, Japan; 6 Drug Discovery Technology 3, Laboratory for Innovative Therapy Research, Shionogi, Osaka, Japan

**Keywords:** Neuroligin 4X, Proteolysis, Trafficking, Synaptogenesis

## Abstract

**Background:**

Several genetic alterations, including point mutations and copy number variations in *NLGN* genes, have been associated with psychiatric disorders, such as autism spectrum disorder (ASD) and X-linked mental retardation (XLMR). *NLGN* genes encode neuroligin (NL) proteins, which are adhesion molecules that are important for proper synaptic formation and maturation. Previously, we and others found that the expression level of murine NL1 is regulated by proteolytic processing in a synaptic activity-dependent manner.

**Methods:**

In this study, we analyzed the effects of missense variants associated with ASD and XLMR on the metabolism and function of NL4X, a protein which is encoded by the *NLGN4X* gene and is expressed only in humans, using cultured cells, primary neurons from rodents, and human induced pluripotent stem cell-derived neurons.

**Results:**

NL4X was found to undergo proteolytic processing in human neuronal cells. Almost all NL4X variants caused a substantial decrease in the levels of mature NL4X and its synaptogenic activity in a heterologous culture system. Intriguingly, the L593F variant of NL4X accelerated the proteolysis of mature NL4X proteins located on the cell surface. In contrast, other variants decreased the cell-surface trafficking of NL4X. Notably, protease inhibitors as well as chemical chaperones rescued the expression of mature NL4X.

**Limitations:**

Our study did not reveal whether these dysfunctional phenotypes occurred in individuals carrying *NLGN4X* variant. Moreover, though these pathological mechanisms could be exploited as potential drug targets for ASD, it remains unclear whether these compounds would have beneficial effects on ASD model animals and patients.

**Conclusions:**

These data suggest that reduced amounts of the functional NL4X protein on the cell surface is a common mechanism by which point mutants of the NL4X protein cause psychiatric disorders, although different molecular mechanisms are thought to be involved. Furthermore, these results highlight that the precision medicine approach based on genetic and cell biological analyses is important for the development of therapeutics for psychiatric disorders.

## Introduction

Autism spectrum disorder (ASD) is a neurodevelopmental disorder defined by impaired social interactions, communication deficiency, restricted interests, and stereotyped activity patterns. Various genetic and environmental factors have been implicated in the pathogenesis of ASD, although the precise mechanism remains unclear [1]. Recently, several studies identified many types of genetic variations associated with ASD patients. Among them, several nonsense, missense, and deletion mutations were found in human *NLGN* genes, primarily in patients with ASD. *NLGN* genes encode neuroligin (NL) proteins, which are postsynaptic adhesion molecules involved in the formation and plasticity of synapses with neurexins, which are presynaptic ligands [2–4]. The human NL family comprises NL1, NL2, NL3, NL4X, and NL4Y. In rodents, NL4*, which shows 57% homology to NL4X, is expressed instead of NL4X and NL4Y. NL1 specifically localizes at excitatory synapses, whereas NL2 and NL4* are found at inhibitory synapses. NL3 is targeted to both synapses. Intriguingly, several copy number variations and protein truncation mutations were identified in the *NLGN4X* gene of autistic patients [5–8], suggesting that loss-of-function of NL4X underlies the pathomechanisms of ASD caused by the *NLGN4X* gene. Moreover, several point mutations associated with autistic patients in the *NLGN4X* and *NLGN4Y* genes have been identified [9–12]. In addition to ASD, recent advances in genome analyses have demonstrated the presence of missense variations in the *NLGN4X* gene in families of patients with X-linked mental retardation (XLMR) [13–15]. Among them, the R87W substitution was found to impair glycosylation of NL4X, thereby causing its retention in the endoplasmic reticulum, resulting in inactivation of synaptogenic function [12]. Recently, the R704C variant of NL4X has been identified as a change-of-function mutation in human neurons [16]. However, the molecular biological effect(s) of the other variants remains unclear to date.

We and others have demonstrated that NL1 undergoes synaptic activity-dependent proteolytic processing [17, 18]. Specifically, the NL1 protein on the cell surface is cleaved by a disintegrin and metalloproteinase domain-containing protein 10 (ADAM10) and matrix metalloproteinase 9 at the proximal region to the membrane, to release a soluble extracellular domain of NL1 (sNL1). The remaining C-terminal fragment is then processed by γ-secretase and degraded. This cleavage of NL1 determines the cell-surface level of functional NL1, thereby affecting its synaptogenic function. Other NLs also undergo proteolytic processing and are implicated in the pathology of ASD [19]. However, the processing of human NL4X has not been analyzed to date. In addition, the association between the proteolytic processing and disease-associated variants in *NLGN4X* has remained unknown. Here, we found that aberrant protein maturation and processing of NL4X are involved in the pathomechanisms of *NLGN4X*-associated ASD and XLMR. Correction of NL4X protein metabolism by small compounds is hence expected to be a promising novel therapeutic strategy against ASD and XLMR.

## Materials and methods

### Expression vectors and culturing cells

All experimental procedures were performed in accordance with the guidelines for animal experiments and human samples of the University of Tokyo as well as Keio University. cDNA expressing HA-tagged NL4X provided by Dr. Peter Scheiffele [20] was subcloned into pcDNA3.1 directional TOPO expression vector to generate a vector encoding HA-NL4-V5/His [17]. cDNAs encoding NL4X variant were generated by long PCR-based mutagenesis and analyzed by sequencer. Maintenance of COS-1 cells, HEK293 cells, IMR32 cells, and embryonic fibroblasts derived from ADAM knock-out mice of either sex [21–24] were described previously [17]. Primary cortical cultures were prepared from the brains of embryonic day (E) 15-17 or postnatal day (P) 1 Balb/C mice or E17-18 Wistar rats as previously described [17, 25, 26]. Briefly, dissociated neurons were plated at 2.6 × 10^5^ cells per cm^2^ on plates coated with poly-l-ornithine (SIGMA) and cultured in DMEM high glucose (Wako) supplemented with 50 unit/ml penicillin, 50 mg/ml streptomycin (Invitrogen), 0.25 μg/ml plasmocin (InvivoGen), and 10% FBS (HyClone). On the following day, the cultured medium was replaced with Neurobasal medium (Invitrogen) supplemented with 2 mM l-glutamine, 50 unit/ml penicillin, 50 mg/ml streptomycin, 0.25 μg/ml plasmocin, and B-27 supplement (Invitrogen). Cultures were maintained at 37 °C in a 95% air/5% CO_2_ humidified incubator, and half of the medium was changed every 3 or 4 days before use. Coculture of NL4X-expressing HEK293 cells and the primary neurons was performed as previously described [4, 26].

### Culture and neuronal differentiation of human induced pluripotent stem cells (iPSCs)

Human ethics approval for experiments using healthy control human iPSCs was obtained from the Ethics Committee in Keio University School of Medicine (approval number 20080016). The healthy control human iPSC line WD39 [27] was cultured in StemFit AK02N (Ajinomoto) on 6-well plates coated with iMatrix-511 (Nippi). Cortical neuron induction of iPSCs was performed according to the prior literatures with slight modifications [28–30]. Briefly, semiconfluent iPSCs were cultured for 14 days in medium hormone mix (MHM) [31–33] with selected growth factors and inhibitors: the growth factors and inhibitors included B27 supplement (Invitrogen), 2 μM SB431542 (Tocris), 0.5 μM LDN193189 (StemRD), and 1.5 μM IWP-2 (Sigma) for the first week, and B27 supplement, 150 nM LDN193189, and 1.5 μM IWP-2 for the second week. For the induction of inhibitory neurons, the growth factors and inhibitors included B27 supplement (Invitrogen), 2 μM SB431542, 0.5 μM LDN193189 1.5 μM IWP-2, and 1 μM purmorphamine (Calbiochem) for the first week, and B27 supplement, 2 μM SB431542, 1.5 μM IWP-2, and 1 μM purmorphamine for the second week. The consequent neural progenitor cells were dissociated and seeded at a density of 5 × 104 cells/cm^2^ on 24-well plate coated with poly-ornithine and laminin. Terminal differentiation was induced in MHM supplemented with B27, 10 μM forskolin (Sigma), and 10 μM DAPT (Sigma) for 5 days. After day 6, the culture was maintained in neural medium (Neurobasal/B27 supplemented with 10 ng/mL BDNF, 10 ng/mL GDNF, 200 μM ascorbic acid, 0.5 mM dbcAMP), and changed medium every 3–4 days with a half volume until day 56. For the last 3 days, the cultures were incubated with 0.1% DMSO or 10 μM INCB3619 in neural medium. Here, we defined the day on which terminal differentiation was started as day 0.

### Generation of NL4X knock-out iPSC-derived neural cells

Studies with human Ngn2-knock-in (KI) iPSCs were approved by the Ethics Committee on Human Tissue and Genome Research at Shionogi & Co., Ltd. (approval number KS17-027, KS18-016). Feeder-free 201B7 human iPSCs [34, 35] were purchased from iPS Academia Japan Inc. Ngn2 KI iPSCs were generated as described in [36]. NL4X knock-out (KO) iPSC clone was generated as follows. Briefly, an all-in-one vector which contains gRNA and Cas9 and pCXN-EGFP vector were electroporated into Ngn2-KI iPSCs using NEPA21 (Nepagene). Targeted sequence of gRNA was 5′- AAGAACACCGTTACCCAATG-3′ and was designed to lower risks of off-target using CRISPR direct (https://crispr.dbcls.jp/). After 2 days of electroporation, GFP+ cells were sorted by FACS aria III (BD) and cloned in 96 well plates. After expansion of cloned cells, sequences of both alleles of each clone were confirmed, and a clone whose both sequences were frame-shifted was used as an NL4X KO iPSC clone.

Sequence of NLGN4X (169-195)

18WT: GGCCTAAGAACACCGTTACCCAATGAG

Mut a: GGCCTAA----------------TGAG (-16)

Mut b: GGC-------------TACCCAATGAG (-13)

Differentiation of iPSCs to neural progenitors (NPs) and neurons in adherent culture was performed as described in [37] with slight modification. Briefly, on day 0, confluent iPSCs were passaged onto Matrigel-coated dishes and cultured in AK03N medium (Ajinomoto). On day 1, doxycycline was added into the medium to induce expression of Ngn2. On day 2, an equal volume of N2 medium was added to the AK03N medium, and N2 medium was used on days 3–4. On day 5, an equal amount of NB medium was added to the N2 medium, and NB medium was used on days 6–8. From day 5, cytosine arabinoside (AraC) was added to the medium to inhibit proliferation of NPs. N2 medium contains DMEM/F12, 1X N2 supplement (Invitrogen), 1X NEAA (Invitrogen), mouse laminin (0.2ug/mL), NT-3 (10 ng/mL), and BDNF (10 ng/mL). NB medium contains Neurobasal, 1X B-27 supplement (Invitrogen), 1X GlutaMAX-I supplement (Invitrogen), mouse laminin (0.2ug/mL), NT-3 (10 ng/mL), and BDNF (10 ng/mL). From day 11, BrainPhys Neuronal Medium and SM1 supplement (Stem Cell Technology) were used to promote further neural maturation.

### Antibodies and compounds

The following antibodies were used: HA-high (3F10, Roche, ×2000 dilution), α-tubulin (DM1A, SIGMA, ×2000 dilution), βIII-tubulin (TUJ1, SIGMA, ×5000 dilution), VGAT (#131002, Synaptic Systems, ×1000 dilution), vGlut1 (#135303, synaptic systems, ×1000 dilution), V5 tag (R960-CUS, Invitrogen, ×5000 dilution) ADAM10 (ab1997, abcam, ×500 dilution). For rabbit polyclonal antibody, SAJ520206 was raised against synthetic peptide corresponding to NL4X cytoplasmic region (723-741) by SIGMA. For rat monoclonal antibody against extracellular region of NL4X, we injected 250 μg of the recombinant human NL4X protein (5158-NL, R&D Systems) with Freund’s adjuvant complete (SIGMA) into the foot pad of WKY/Izm rat. After three additional immunization with Freund’s adjuvant incomplete (SIGMA), iliac and inguinal lymph nodes were obtained. B cells were fused with PAI cells (JCRB0113) by polyethylene glycol (Roche) and cultured with GIT medium containing 5% FBS, hypoxanthine/aminopterin/thymidine (SIGMA), and BM Condimed H1 (Roche). Screening was performed by immunocytochemical analysis using HEK293 cells stably expressing NLs. After limiting dilution and further screening, we selected clone 2C3 as human NL4X specific rat monoclonal antibody. 4PBA was purchased from SIGMA. INCB3619 was synthesized according to the patent descriptions as previously described [17].

### Immunological analyses

Immunoblotting was performed as described previously [38]. Briefly, cell lysates were collected with sample buffer (2% SDS, 80 mM Tris-HCl pH 6.8, 10% glycerol, Brilliant green (WAKO), Coomassie blue G-250 (Nacalai tesque)). Protein concentrations of the samples were determined by BCA protein kit (Thermo Fisher Scientific). Then, the samples were boiled at 100° for 3 min after addition of 1% 2-mercaptoethanol (WAKO). Same amount of proteins (typically, 10 μg/lane) were separated by SDS-PAGE, transferred onto the PVDF membrane (Millipore). After incubation of the membranes with appropriate antibodies conjugated with fluorescent dyes, bands were detected using Image Quant LAS4000 (GE Healthcare). Band intensities were quantified by ImageJ. Cell surface biotinylation assay using Sulfo-NHS-LC-biotin (Pierce) was performed as previously described [17].

For detection of soluble form of NL4X produced from IMR32 cells and iPSC neurons, the cultured medium was immunoprecipitated using 2C3 antibody. After centrifugation to remove the cell debris, the conditioned medium was incubated with rat IgG or 2C3 antibody at 4° for overnight on a rotator. Proteins interacted with antibodies were precipitated by protein G sepharose beads (GE Healthcare) and analyzed by immunoblotting.

Human brain sample was derived from tissue bank at the University of Pennsylvania Alzheimer’s Disease Core Center (ADCC) and the Center for Neurodegenerative Disease Research (CNDR) [39]. All samples used for experimental measures were derived from the frontal cortex under approval by the institutional review board, ADCC–CNDR, and institutional ethical committee of Graduate School of Pharmaceutical Sciences, The University of Tokyo (No. 12-1). Tris buffer (TS; 50 mM Tris HCl, pH 7.6, 150 mM NaCl, 0.5 mM diisopropyl fluorophosphate, 0.5 mM phenylmethylsulfonyl fluoride, 1 mM EGTA, 1 mg/ml antipain, 1 mg/ml leupeptin, 1 mg/ml pepstatin, 1 mg/ml Na-p-tosyl-l-lysine chloromethyl ketone) soluble and insoluble fractions from control brain were used [40]. The insoluble fraction was solubilized by TS containing 1% Triton X-100 (Tx) and analyzed after centrifugation.

### Immunocytochemical analyses

Samples were fixed by 4% PFA containing PBS and stained as previously described [41]. Briefly, fixed cells were permeabilized by 0.1% Triton X-100 and incubated with primary antibody as indicated for 2 h. After washing with PBS, cells were incubated with secondary antibody for 30 min and mounted on slide glass. Samples were observed with a fluorescence microscope (Axio observer Z1, Zeiss, Germany) or a confocal microscope (TCS-SP5, Leica, Germany).

### Quantitative immunofluorescence analyses

Synapse formation assay was performed as described previously [26, 42, 43]. Dissociated cortical neuron was plated onto poly l-lysine-treated glass coverslips at a density of 130 cells/mm^2^. Transfected HEK293 cells were added to primary neuron at 8–10 days in vitro. After 2 days of coculture, cells were fixed then performed immunocytochemical analysis. Synapse formation was quantified as the average fluorescence intensity of VGAT positive puncta over HA-tagged NL4X transfected HEK293 cells. Images were acquired in a blind manner to experimental condition using laser scanning microscope (TCS-SP5, Leica). Using the ImageJ software, the averaged immunofluorescent signals per total area were obtained and normalized to the mean values of control experiments.

### Statistical analyses

Statistical tests were indicated at each figure legends. All data are presented as mean ± SEM.

## Results

### Metabolism of the NL4X protein in human tissue and cultured cells

**Fig. 1:**
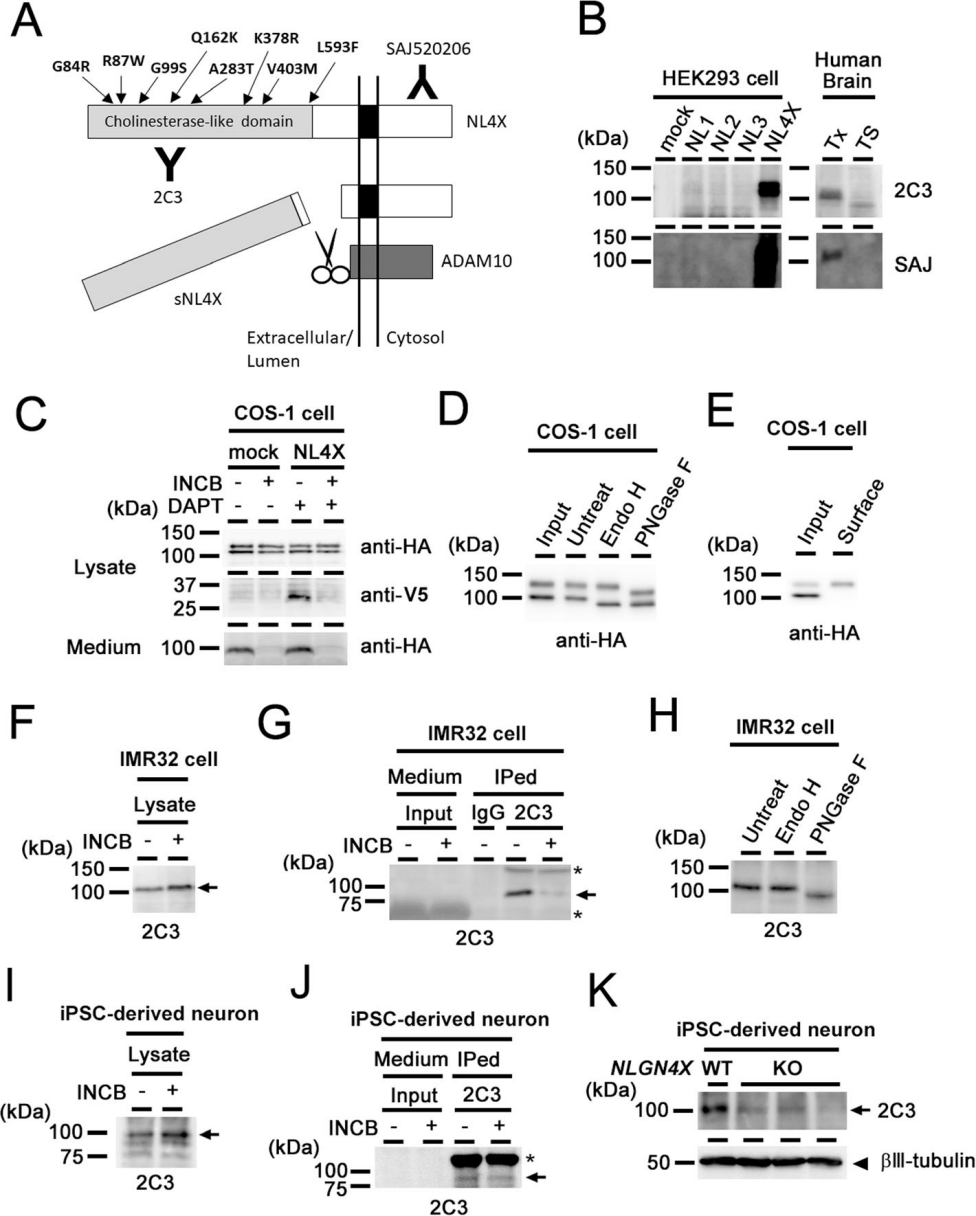
Metabolism of NL4X protein. **a** Schematic depiction of NL4X metabolism. Positions of ASD/XLMR-associated variants are indicated by arrow. Epitopes of antibodies are also shown. **b** Characterization of antibodies used in this study. **c** Protein metabolism of overexpressed NL4X in COS-1 cells. **d** Deglycosylation assay of overexpressed NL4X in COS-1 cells. **e** Cell surface biotinylation of overexpressed NL4X in COS-1 cells. **f** Detection of endogenous NL4X in IMR32 cells. **g** Immunoprecipitation of endogenous sNL4X secreted from IMR32 cells. **h** Deglycosylation assay of endogenous NL4X in IMR32 cells. **i** Detection of endogenous NL4X in iPSC-derived human neurons. **j** Immunoprecipitation of endogenous sNL4X secreted from iPSC-derived human neurons. **k** Immunoblot analysis of the lysates of the NL4X KO iPSC-derived human neurons

We previously showed that murine NL1 and NL2 undergo sequential proteolysis mediated by metalloprotease-dependent cleavage to release their soluble ectodomains, and the C-terminal stub is then processed by γ-secretase [17]. However, the metabolic pathway of the human NL4X protein remains unclear to date. To clarify this point, we established rat monoclonal and rabbit polyclonal antibodies against the recombinant human NL4X ectodomain (clone 2C3) and against a synthetic peptide corresponding to its intracellular region (SAJ520206). To test the specificity of these antibodies, we overexpressed the wild-type (WT) NL4X protein with N-terminal HA and C-terminal V5-His tags (Fig. 1a). These antibodies specifically recognized the overexpressed HA-NL4X-V5His as 110-120 kDa doublet bands in the HEK293 as well as COS-1 cell lysates on immunoblot analysis (Fig. 1b, c). In addition, 100–110 kDa bands were detected in Triton-soluble (Tx) lysates of the human brain by these different anti-NL4X antibodies raised against distinct regions (i.e., 2C3 against extracellular domain, SAJ520206 against intracellular domain of NL4X). Furthermore, 2C3 antibody recognized the 100 kDa singlet band in the lysates of IMR32 human neuroblastoma cell line and human induced pluripotent stem cell (iPSC)-derived neurons (Fig. 1b, f, i). We then examined the reactivity of 2C3 antibody in the lysates of NL4X knock out (KO) iPSC-derived neurons. The levels of band reacted with 2C3 antibody were decreased in NL4X KO iPSC-derived neurons (Fig. 1k). Correctively, these data indicated that 2C3 antibody mainly reacts with endogenous human NL4X.

The deglycosylation assay using endoglycosidase H and peptide-*N*-glycosidase F demonstrated that the 120-kDa form of NL4X contains complex-type *N*-glycans that are attached to the protein in the Golgi, whereas the 100-kDa form of NL4X was modified only by high mannose-type *N*-glycans, which are attached in the endoplasmic reticulum (Fig. 1d). In contrast, almost all endogenous NL4X in IMR32 cells also contains complex-type *N*-glycans (Fig. 1h). Moreover, the cell-surface biotinylation assay indicated that the 120-kDa form of NL4X was specifically detected at the cell surface (Fig. 1e). In fact, endogenous NL1 and NL2 in primary neuron were observed as singlet band, although overexpressed NL1 and NL2 in COS-1 cells appeared as doublet bands [17], suggesting that the overexpression of membrane protein caused a delay in the protein maturation. These data suggested that 110-kDa NL4X (i.e., immature NL4X) undergoes complex-type *N*-glycosylation during its trafficking through the secretory pathway, and 120-kDa NL4X (i.e., mature NL4X) is displayed on the cell surface.

Next, we analyzed the proteolytic processing of overexpressed NL4X. In the conditioned medium and cell lysate of COS-1 cells expressing NL4X, we found the HA-tagged 95-kDa soluble NL4X (sNL4X) and the V5-tagged 30-kDa C-terminal fragment, respectively, and the latter accumulated by treatment with the γ-secretase inhibitor (Fig. 1c). In addition, the secretion of sNL4X as well as the accumulation of the C-terminal fragment was reduced by the ADAM inhibitor INCB3619 (Fig. 1c), suggesting that NL4X is sequentially cleaved by metalloproteases and γ-secretase in a similar manner to NL1 [17]. Endogenous sNL4, which was diminished by INCB3619 treatment, was also detected in the conditioned medium of human neuroblastoma IMR32 cells and human iPSC-derived forebrain cortical neurons (Fig. 1g, j). Furthermore, we detected 95 kDa sC3-positive band in the Tris buffer (TS) soluble fraction of human brain, suggesting that NL4 shedding occurs in human neurons.

**Fig. 2:**
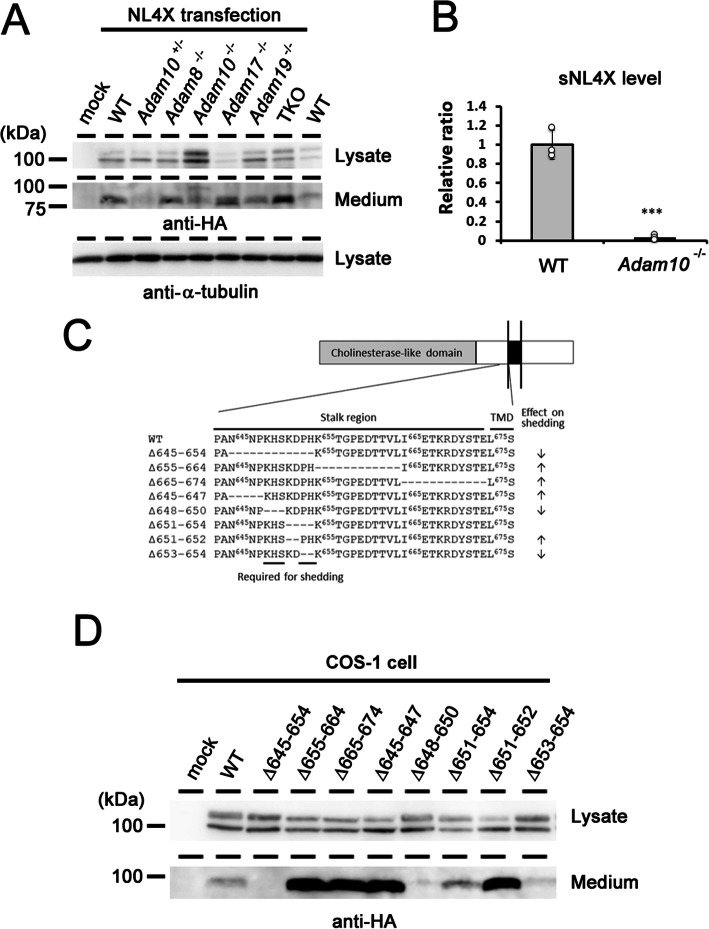
Characterization of NL4X shedding. **a** Immunoblot analysis of overexpressed NL4X in various fibroblasts derived from *Adam* KO mice. **b** Quantification of the levels of sNL4X in the conditioned medium from *Adam10* KO fibroblasts (*n* = 3, ***p < 0.001 vs WT by student *t* test). **c** Schematic depiction of NL4X deletion mutants analyzed in this study. **d** Immunoblot analysis of overexpressed NL4X mutants. Note that several mutants affected the shedding of NL4X as summarized in **c**

To identify the protease responsible for sNL4X production, we overexpressed NL4X in murine embryonic fibroblasts obtained from several ADAM-knockout mice (Fig. 2a). The production of sNL4X was abolished by the genetic ablation of *Adam10*, indicating that ADAM10 is a major protease that cleaves NL4X (Fig. 2b). We then systematically mutated the stalk region of NL4X near the transmembrane domain (Fig. 2c). Based on the molecular weight of sNL4X, we focused on the region corresponding to N645 to E674. Deletion of N645 to H654 abolished the production of sNL4X, whereas deletion of K655 to L664 and I665 to E674 increased the cleavage. Furthermore, deletion of K648/H649/S650 or P653/H654 inhibited the production of sNL4X. In contrast, deletion of N645/N646/P647 or K651/D652 significantly augmented the cleavage, suggesting that these residues are crucial for the cleavage by ADAM10 (Fig. 2c). Collectively, these data indicate that the NL4X protein also undergoes endoproteolysis by ADAM10 at the stalk region to produce sNL4X.

### ASD/XLMR-associated missense variants impaired the function, trafficking, and processing of NL4X

**Fig. 3:**
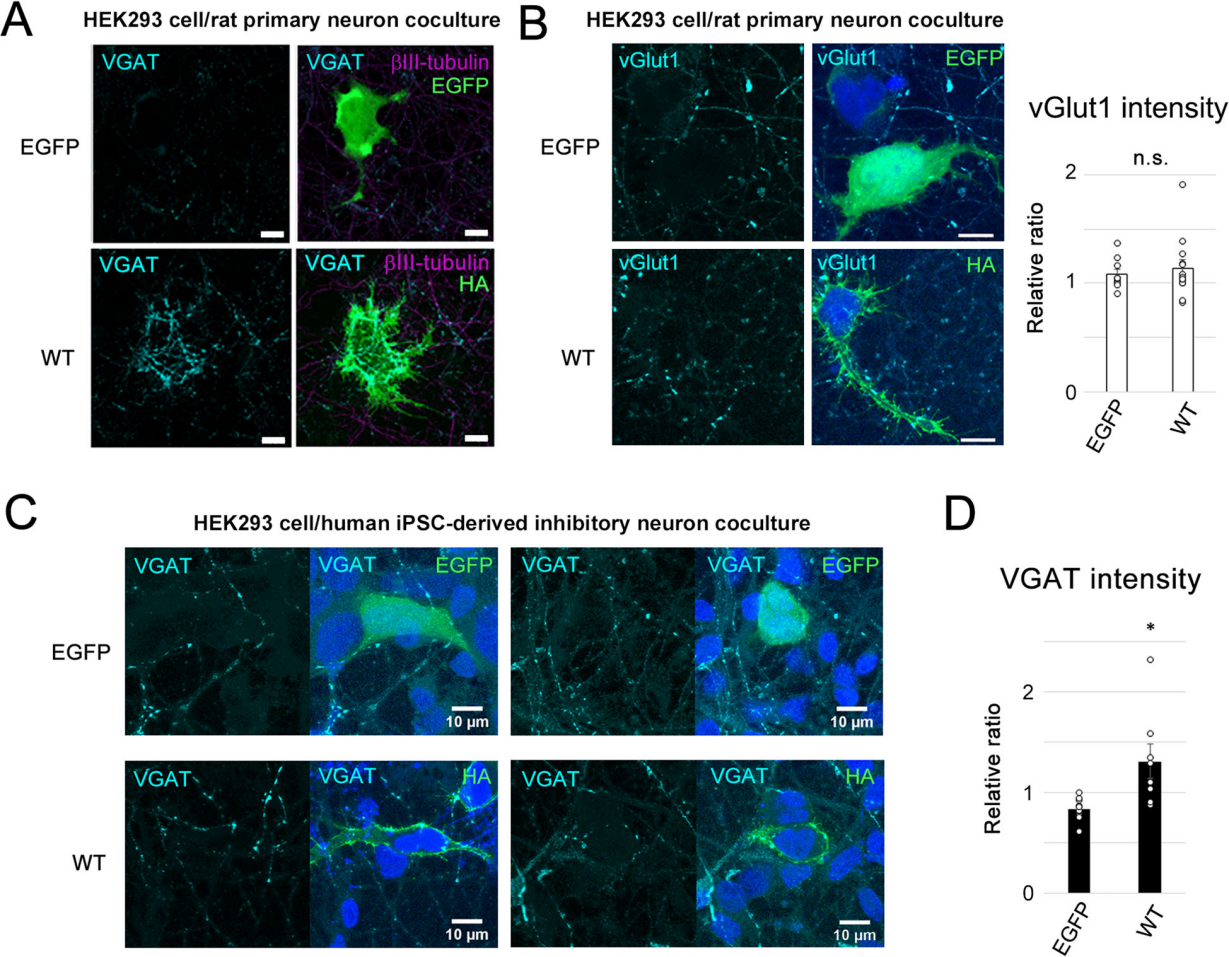
Synaptogenic activity of NL4X in rat primary neurons and human iPSC-derived inhibitory neurons. **a** Representative images of synaptogenic activity of WT NL4X expressed in HEK293 cells cocultured with rat primary neurons. Formation of inhibitory presynapse was visualized by immunostaining using anti-VGAT antibody. Quantification of the intensity of VGAT puncta was shown in Fig. 4a. Scale bar 10 μm. **b** Formation of excitatory presynapse was visualized by immunostaining using anti-vGlut1 antibody in the coculture of NL4X-expressing HEK293 cells and rat primary neurons. Quantification of vGlut1 puncta was shown at right (*n* = 10 (EGFP), 13 (WT), Welch’s *t* test; n.s., not significant). **c** Representative images of synaptogenic activity of WT NL4X expressed in HEK293 cells cocultured with human iPSC-derived inhibitory neurons. **d** Quantification of VGAT puncta in human iPSC-derived inhibitory neurons (*n* = 9 (EGFP), 8 (WT), *p < 0.05 vs WT by Welch’s *t* test)

**Fig. 4:**
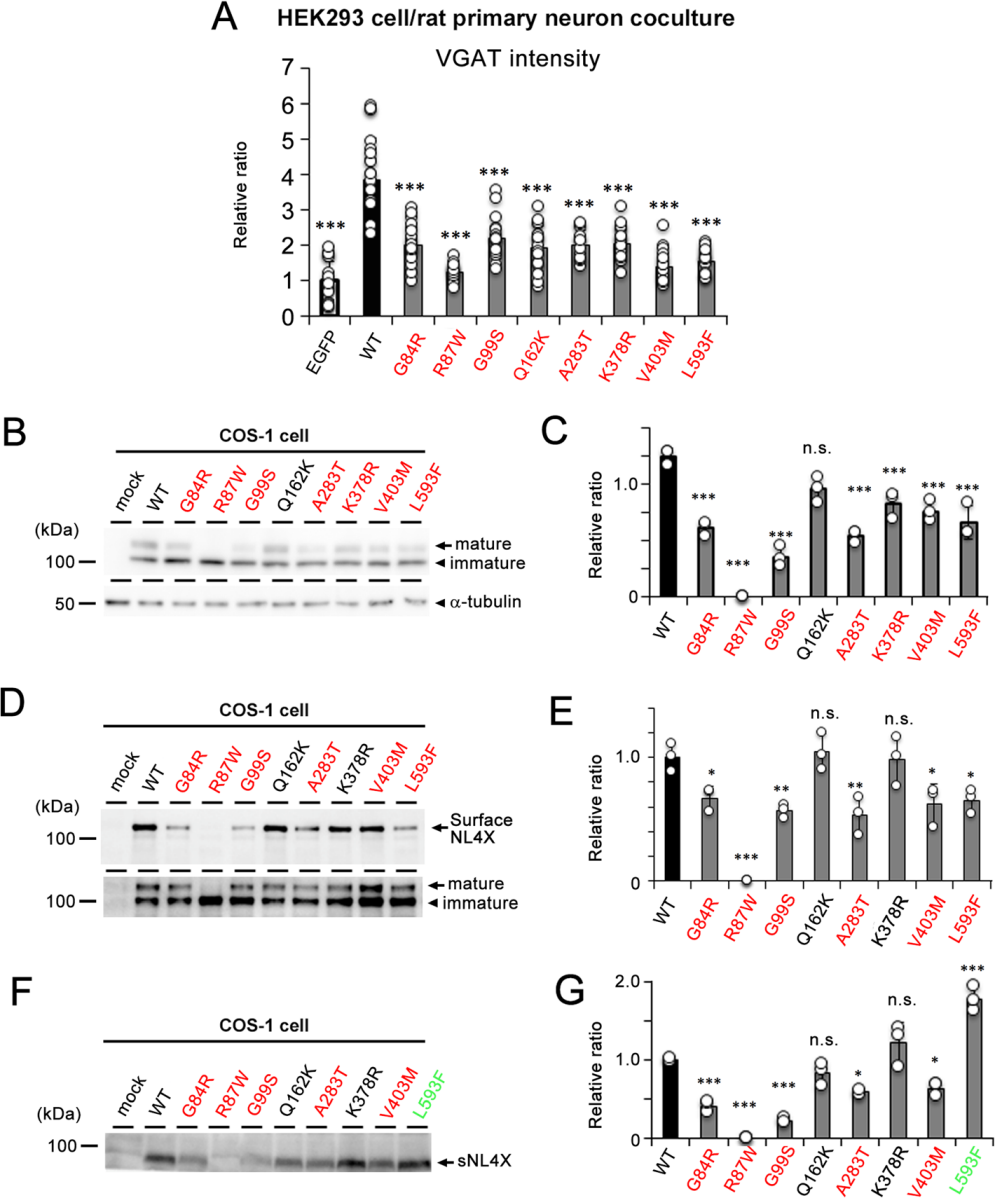
Effect of ASD/XLMR associated NL4X variants on the synaptogenic activity and the protein metabolism. **a** Quantitative result of synaptogenic activity of NL4X variants. Intensity of VGAT puncta was counted and standardized by the area of HEK293 cells (*n* = 12–17, ***p < 0.001 vs WT by Tukey-Kramer multiple comparisons test). **b** Representative immunoblot of cell lysates of COS-1 cells transfected with missense variants of NL4X. **c** Quantitative analysis of the ratio of mature NL4X (*n* = 3, ***p < 0.001 vs WT by one-way ANOVA followed by Dunnett test; n.s., not significant). **d** Representative immunoblot of biotinylated cell surface NL4X variants. **e** Quantitative analysis of the western blot for biotinylated WT and variants (*n* = 3, *p < 0.05, **p < 0.01, ***p < 0.001 vs WT by one-way ANOVA followed by Dunnett test; n.s. not significant). **f** Representative immunoblot of conditioned medium from COS-1 cells transfected with missense variants of NL4X. **g** Quantitative analysis of the western blot for sNL4X (*n* = 3, *p < 0.05, **p < 0.01, ***p < 0.001 vs WT, by one-way ANOVA followed by Dunnett test; n.s., not significant)

To analyze the effects of ASD/XLMR-associated missense variants on the synaptogenic activity of NL4X, we analyzed synapse formation using a heterologous culture assay [44]. Because coculture of COS-1 cells caused neurotoxicity in our laboratory [17], we utilized HEK293 cells for expressing NL4X. As mouse NL4*, an orthologue of NL4X, is localized mainly at inhibitory synapses [45, 46], we utilized staining of vesicular GABA transporter (VGAT), an inhibitory presynaptic marker, to assess the formation of inhibitory presynapses by NL4X. Coculture of rat primary neurons and HEK293 cells expressing NL4X induced the accumulation of VGAT puncta around HEK293 cells (Figs. 3a, 4a). In contrast, the intensities of vesicular glutamate transporter 1 (vGlut1), an excitatory synapse-specific protein, were unaltered (Fig. 3b). Moreover, coculture of NL4X-expressing HEK293 cells with human iPSC-derived inhibitory neurons induced the accumulation of VGAT puncta around HEK293 cells (Fig. 3c, d), indicating that NL4X has the ability to form inhibitory presynaptic structures in rat and human neurons.

**Fig. 5:**
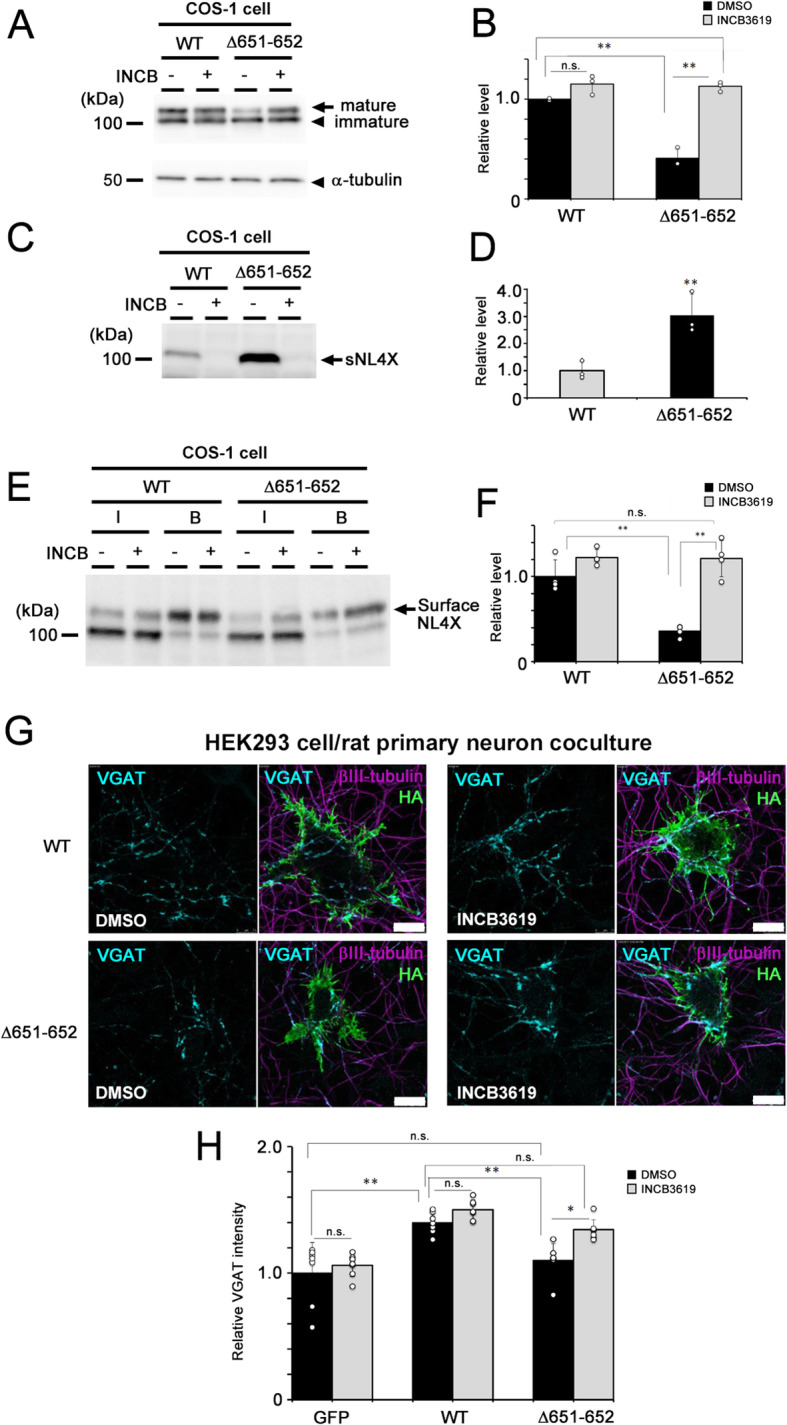
Protein metabolism and synaptogenic function of NL4X Δ651-652 mutant. **a** Representative immunoblot of cell lysates of COS-1 cells transfected with NL4X Δ651-652 mutant. **b** Quantitative analysis of the western blot for mature NL4X (*n* = 3, **p < 0.01 vs WT by Tukey-Kramer multiple comparisons test). **c** Representative immunoblot of conditioned medium from COS-1 cells transfected with NL4X Δ651-652 mutant. **d** Quantitative analysis of the western blot for sNL4X (*n* = 4, **p < 0.01 vs WT, by Student *t* test). **e** Representative immunoblot of biotinylated cell surface NL4X Δ651-652 mutant. **f** Quantitative analysis of the western blot for biotinylated WT and variants (*n* = 4, **p < 0.01 vs WT by Tukey-Kramer multiple comparisons test). **g** Representative images of synaptogenic activity of NL4X Δ651-652 mutant in HEK293 cells. Scale bar 10 μm. **h** Quantitative result of synaptogenic activity of NL4X Δ651-652 mutant (*n* = 4–6, *p < 0.05, **p < 0.01 vs WT by Tukey-Kramer multiple comparisons test)

We then analyzed the effects of ASD/XLMR-associated missense variants of *NLGN4X* on inhibitory synaptogenesis in the coculture of HEK293 cells and rat primary neurons. Intriguingly, all missense variants reduced the formation of VGAT puncta, suggesting that these point mutations caused a loss-of-function in terms of the synaptogenic activity of NL4X (Fig. 4a). Because NLs induce presynaptic structures by their interaction with cognate ligands (e.g., neurexins) at the synaptic contact site [2, 3], the loss-of-function phenotype of NL4X variants might correlate with their cell-surface expression levels. Supporting this notion, R87W completely abolished the formation of mature NL4X and its cell-surface expression, as previously described [12]. We also found that all ASD variants except for Q162K reduced the ratio of mature NL4X to immature NL4X (Fig. 4b, c). Furthermore, the biotinylation experiment revealed that ASD variants except for Q162K and K378R reduced the levels of cell surface NL4X (Fig. 4d, e). We then analyzed the cleavage of ASD variants (Fig. 4f, g). G84R, R87W, G99S, A283T, and V403M variants of sNL4X had substantially decreased expression levels. The levels of sNL4X harboring the Q162K or K378R variant were almost the same as that of WT NL4X. Unexpectedly, sNL4 production was significantly increased in cells expressing the L593F variant. To confirm whether this increased cleavage inhibited synaptogenic activity, we analyzed the metabolism and function of the K651/D652 deletion-mutant NL4X, which was efficiently cleaved (Fig. 2d, e). As expected, the K651/D652 deletion mutant showed a decreased level of mature NL4X and decreased cell-surface expression of mature NL4X (Fig. 5a–f). Moreover, this mutant failed to induce inhibitory synapses in the coculture system (Fig. 5g, h). These data indicate that not only the cell surface level but also the proteolytic processing of NL4X regulates synaptogenic activity. Thus, the decreased synaptogenic function of NL4X by ASD/XLMR-associated variants might be caused by the disturbance of protein metabolism, thereby decreasing the cell-surface levels of NL4X.

### Improved cell-surface expression of the NL4X variant based on its pathogenic mechanism rescued by the dysfunction of synaptogenesis

**Fig. 6:**
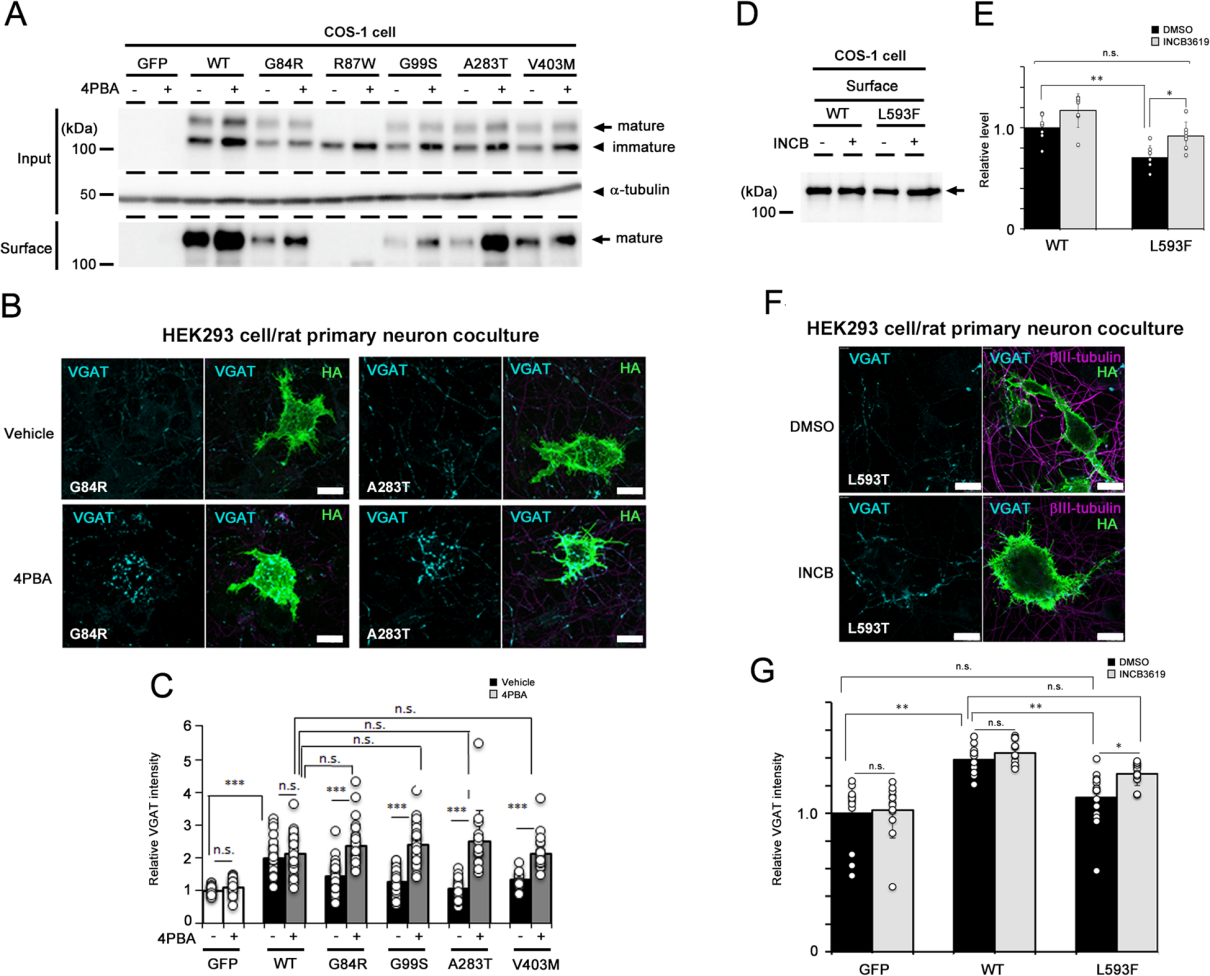
Rescue of synaptogenic function of NL4X variants. **a** Effect of 4PBA on the expression of NL4X variants that showed the impaired maturation. **b** Representative images of synaptogenic activity of NL4X variants treated with 4PBA. Scale bar 10 μm. **c** Quantitative result of synaptogenic activity of NL4X variants treated with 4PBA (*n* = 14–37, **p < 0.001 vs WT with vehicle by Tukey-Kramer multiple comparisons test; n.s., not significant). **d** Effect of INCB3619 on the surface expression of NL4X L593F variant expressed in HEK293 cells. **e** Quantitative analysis of the western blot for biotinylated WT and variants (*n* = 7, *p < 0.05, **p < 0.01 vs WT by one-way ANOVA with Tukey HSD post hoc analysis). **f** Representative images of synaptogenic activity of NL4X L593F variant treated with INCB3619. Scale bar 10 μm. **g** Quantitative result of synaptogenic activity of NL4X L593F variant treated with INCB3619 (*n* = 12–16, *p < 0.05, **p < 0.01 vs GFP by Tukey-Kramer multiple comparisons test)

The majority of NL4X variants demonstrated reduced protein maturation and cell-surface trafficking, as well as synaptogenic function. Such defects have been described in several disease-associated mutations in membrane proteins, such as cystic fibrosis transmembrane conductance regulator (CFTR) in cystic fibrosis and bile salt export pump (BSEP) in progressive familial intrahepatic cholestasis type 2 [47, 48]. Of note, treatment with chemical chaperones, which assist protein folding and induce conformational stabilization, improves protein function in the models of these diseases and symptoms in the patients [49]. To test whether the pharmacological chaperone is effective to the ASD/XLMR-associated NL4X variants, we analyzed the effect of 4-phenylbutyrate (4PBA), which has been tested in the rescue for the expression and function of mutant CFTR as well as BSEP [50, 51]. We found that levels of the mature forms of the G84R, G99S, A283T, and V403M variants, but not R87W, were significantly increased by 4PBA treatment (Fig. 6a). We then analyzed the effect of 4PBA treatment on NL4X variants except for R87W in the synapse formation assay using a coculture system. We found that the accumulation of VGAT-positive puncta on NL4X variant-expressing HEK293 cells was significantly recovered by 4PBA treatment, whereas no effect was observed in the coculture using WT NL4X-expressing cells (Fig. 6b, c). These results suggest that correcting the folding of the NL4X protein by a chemical chaperone is a plausible therapy against cases of ASD/XLMR that are caused by protein misfolding.

The L593F variant, which was originally identified from the screening of a XLMR patient family [15], showed the increased production of sNL4X and its reduced cell-surface level, indicating that the L593F variant upregulated the cleavage of NL4X. Thus, inhibition of the proteolytic processing of NL4X may recover its synaptogenic function. In fact, treatment with the ADAM10 inhibitor INCB3619 restored the level of mature NL4X on the cell surface (Fig. 6d, e). We then analyzed the effect of INCB3619 on the defects of synaptogenic function caused by the L593F variant. We found that INCB3619 treatment augmented the accumulation of VGAT puncta on HEK293 cells expressing the NL4X L593F variant (Fig. 6f, g). Consistent with this result, the synaptogenic activity of the K651/D652 deletion mutant was also rescued by INCB3619 treatment (Fig. 5g, h). Taken together, these data indicated that the correction of cell surface level of NL4X protein by small compounds targeting its pathogenic mechanism successfully recovered the dysfunction of presynapse formation caused by the NL4X variants.

## Discussion

In this study, we found that all ASD and XLMR-associated NL4X variants significantly impaired the ability of inhibitory synapse formation, as described for the R87W variant [52]. Most NL4X variants (i.e., G84R, G99S, A283T, V403M) that were analyzed in this study had reduced protein maturation, which was rescued by the chemical chaperone 4PBA. Intriguingly, a similar dysfunctional phenotype correlating with misfolding has been reported for the P89L variant of *NLGN1* and the R215H variant of *NLGN2*, which are associated with autism and schizophrenia, respectively [53, 54]. In general, the protein quality-control system at the endoplasmic reticulum, consisting of molecular chaperones, proteases, and regulatory factors, assists protein folding and trafficking. These variants demonstrate abnormalities in the folding process at the endoplasmic reticulum as well as in the trafficking to the cell surface. Among the NL4X variants, 3 residues (i.e., A283T, K378R, V403M) are embedded within the folded NL4X protein in the crystal structure [55], suggesting that these substitutions affect the association of amino acids within the internal region of the folded NL4X protein. In contrast, G84R, R87W, and G99S are located on the surface of the NL4X protein. Thus, these N-terminal variants might affect the interaction of NL4X with chaperone proteins, which help their folding and stabilization (e.g., BiP, calnexin, and calreticulin), although the precise folding mechanism of NL4X remain unclear.

We also found that the L593F variant of NL4X showed a decrease in the expression level of its mature form, but by a distinct mechanism from the other variants, namely, by increased cleavage. A similar phenotype has been reported in an Alzheimer disease-associated variant of the *TREM2* [56, 57]. Triggering receptor expressed on myeloid cells 2 (TREM2) is cleaved by ADAM10 at the H157-S158 bond, and the H157Y variant found in an Alzheimer disease patient increased its cleavage and caused a loss-of-function phenotype. Here, we identified that ADAM10 is also responsible for the proteolytic processing of NL4X in a similar manner to that of NL1 [17]. Deletion mutation analyses suggested the possibility that K648/H649/S650 variants of NL4X contain a cleavage site that generates sNL4X. Also, several parts of the stalk region were found to regulate the proteolysis. The steric configuration of L593 and these regions remains unknown because the stalk region is not observed in the NL4X crystal structure. However intriguingly, L593 is located within the four-helix bundle, which is involved in the assembly of a noncovalent antiparallel dimer of 2 NL4 molecules [55]. This dimer-inducing bundle structure is common among all NLs, and dimer formation of NL facilitates synaptic assembly [58]. Thus, the L593F substitution might cause dissociation of the NL4X dimer to increase the accessibility of the stalk region to ADAM10, and thereby accelerate the cleavage.

In this study, we were unable to clarify the molecular pathology of the Q162K and K378R variants, although these variants also showed defects in the synapse formation assay. Both residues were not involved in the binding of NL4X with neurexin in the crystal structure [55]. However, these residues might be involved in stabilization of the NL4X complex with neurexin and/or other ligands. In addition, the K378R variant of the NL4X protein is expected to demonstrate an altered subcellular localization and intracellular trafficking, as it showed reduced amounts of its mature form without any effect on its cell-surface level and cleavage. Thus, further investigation would be required for understanding the pathomechanism of Q162K and K378R variants at molecular level.

We tried to rescue the loss-of-function phenotype of NL4X variants by distinct pharmacological approaches based on their molecular pathomechanisms. For variants causing misfolding and ER retention, treatment with the chemical chaperone 4PBA recovered their cell surface level and synaptogenic activity. This chemical chaperone has been shown to be effective against several disease-associated dysfunctional mutations of membrane proteins, including CFTR and BSEP [50, 51, 59]. The use of chemical chaperones/protein correctors has been approved for the treatment of cystic fibrosis and other diseases. Notably, several amino acid substitutions in *NLGN* genes that cause misfolding have been reported, such as P89L in *NLGN1* [54], R215H in *NLGN2* [53], and R451C, P514S, and R597W in *NLGN3* [60, 61]. Thus, a pharmacological approach using chemical chaperones is a plausible therapeutic approach for NL variant-associated psychiatric diseases. Moreover, we found that treatment with a metalloprotease inhibitor was effective against the synaptogenic dysfunction caused by variants that induce accelerated cleavage. Physiologically, cleavage regulates the amount and activity of cell surface NLs [17, 18]. Notably, INCB3619 treatment did not cause any abnormalities in the synaptogenic activity of WT NL4X in our study. However, global inhibition of ADAM10 activity might have detrimental effects because a wide range of substrates for ADAM10 have been reported [62]. Thus, spatiotemporal control of ADAM10 activity in ASD/XLMR patients who express NL4X variants with increased cleavage is required. Nevertheless, we found that disease-associated variants of NLGN4X demonstrate a loss-of-function of synaptogenic activity by a distinct mechanism, namely, protein misfolding and augmentation of cleavage.

That is, our data suggested that maintaining adequate NLGN4X expression level is necessary for neuronal function, and its dysfunction caused psychiatric disorders including ASD/XLMR.

## Limitations

Our study did not reveal whether these dysfunctional phenotypes occurred in individuals carrying *NLGN4X* variant. Moreover, though these pathological mechanisms could be exploited as potential drug targets for ASD, it remains unclear whether these compounds would have beneficial effects on in ASD model animals and patients.

## Conclusions

These data suggest that reduced amounts of the functional NL4X protein on the cell surface is a common mechanism by which point mutants of the NL4X protein cause psychiatric disorders, although different molecular mechanisms are thought to be involved. Furthermore, these results highlight that the precision medicine approach based on genetic and cell biological analyses is important for the development of therapeutics for psychiatric disorders.

## Supplementary information

**Additional file 1:.** Supplemental figures.

## Data Availability

We would like to share any data and materials described in this study.

## Abbreviations

4PBA: 4-Phenylbutyrate
ADAM10: A disintegrin and metalloproteinase domain-containing protein 10
ASD: Autism spectrum disorder
BSEP: Bile salt export pump
CFTR: Cystic fibrosis transmembrane conductance regulator
GFP: Green fluorescent protein
iPSC: Induced pluripotent stem cells
KI: Knock-in
KO: Knock-out
NL: Neuroligin
sNL: Soluble extracellular domain of NL
MHM: Medium hormone mix
TREM2: Triggering receptor expressed on myeloid cells 2
TS: Tris buffer
Tx: Triton-soluble
VGAT: Vesicular GABA transporter
vGlut1: Vesicular glutamate transporter 1
WT: Wild-type
XLMR: X-linked mental retardation
