# Supplementary material for: Autism-associated variants of neuroligin 4X impair synaptogenic activity by various molecular mechanisms
Source: Mol Autism. 2020 Sep 1;11:68. doi: 10.1186/s13229-020-00373-y (PMC7465329; doi:10.1186/s13229-020-00373-y)

# Figure 1B

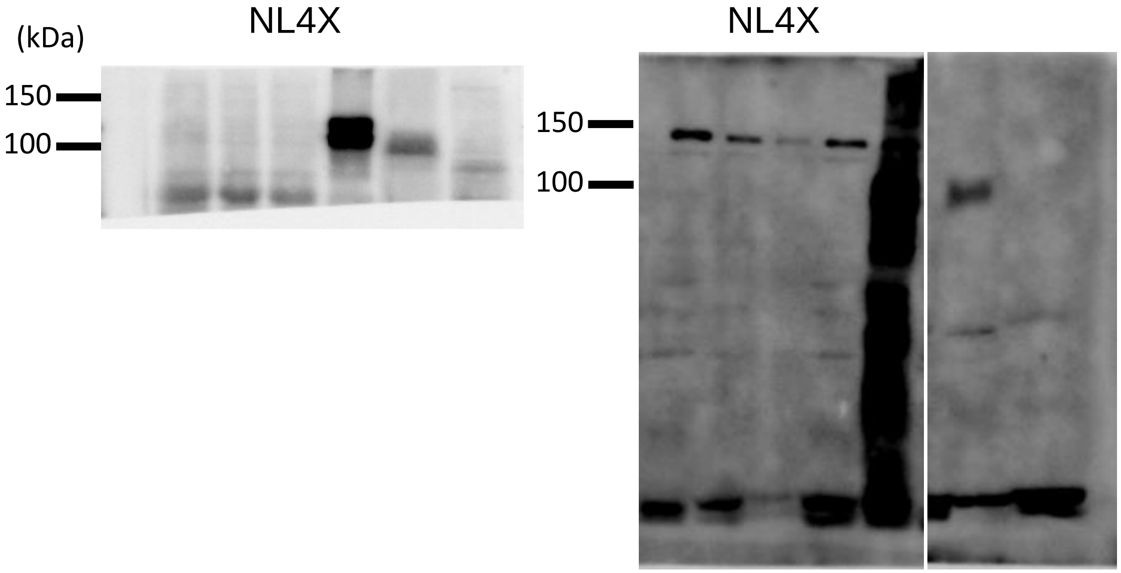


# Figure 1C

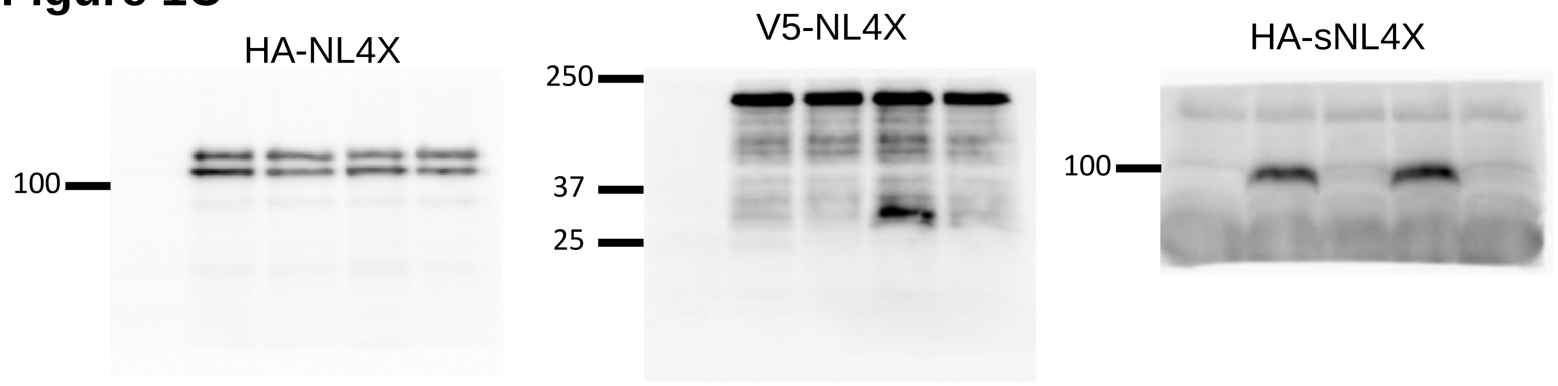

## Figure 1D

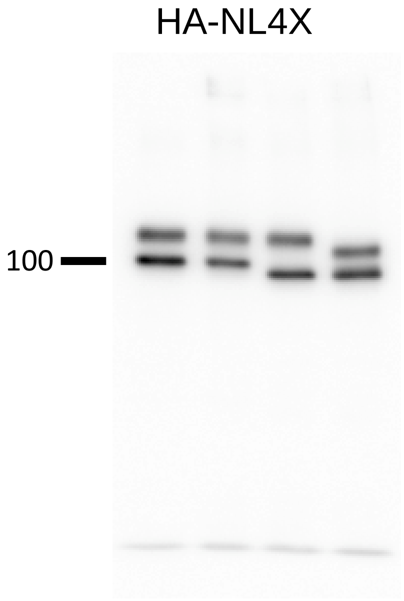


## Figure 1E

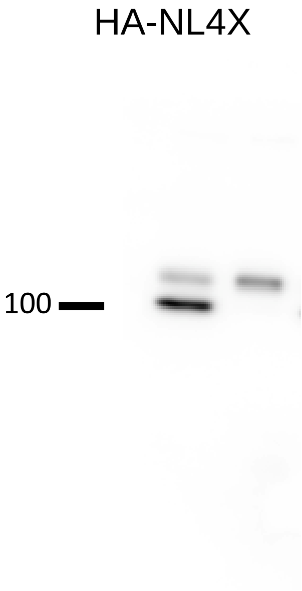


## Figure 1F

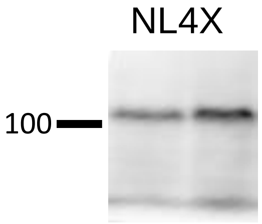


## Figure 1G

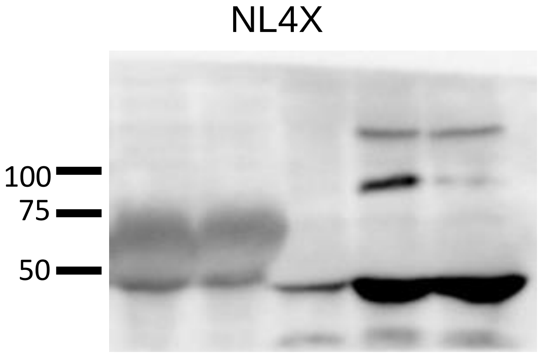


## Figure 1H

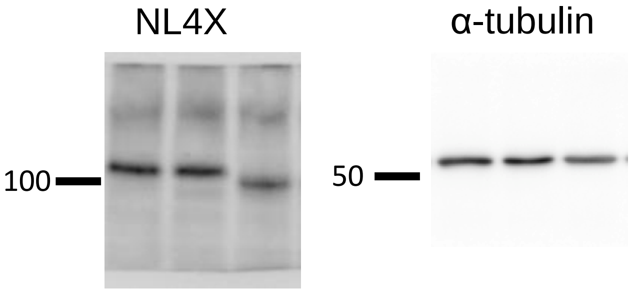


## Figure 1I

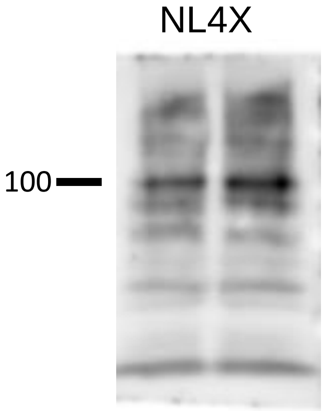


## Figure 1J

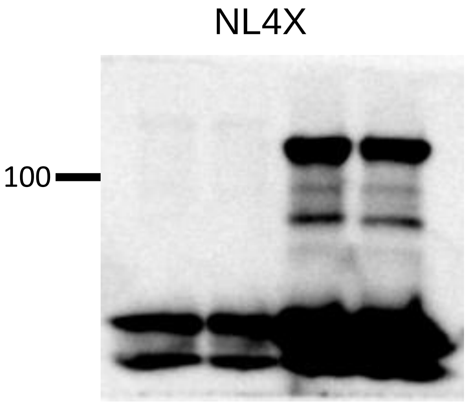

## Figure 1K

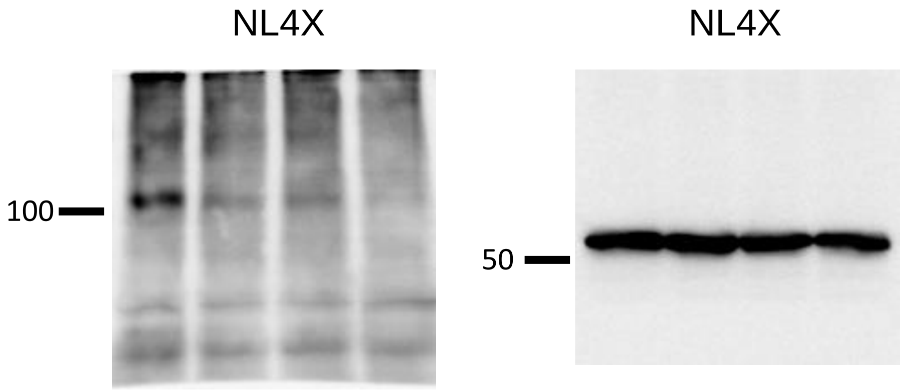


## Figure 2A

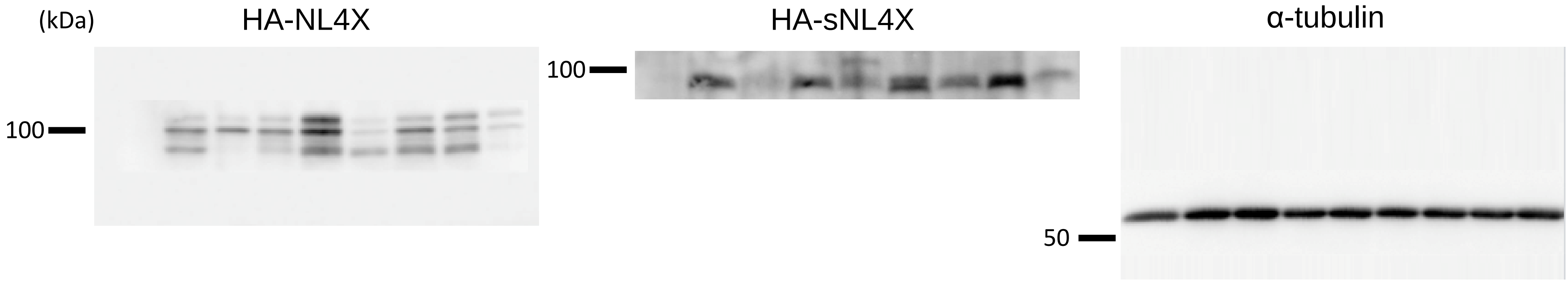

## Figure 2D

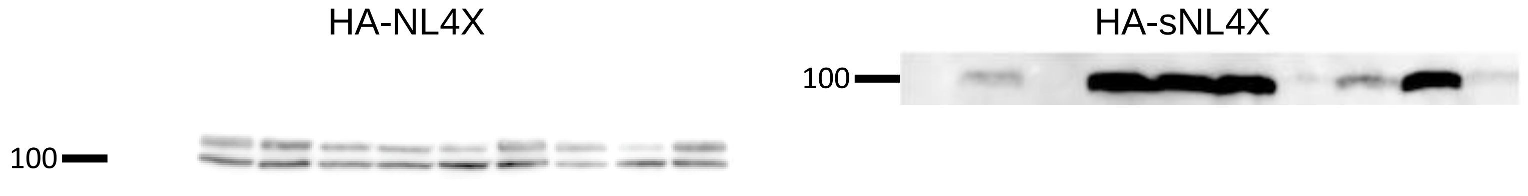


## Figure 4B

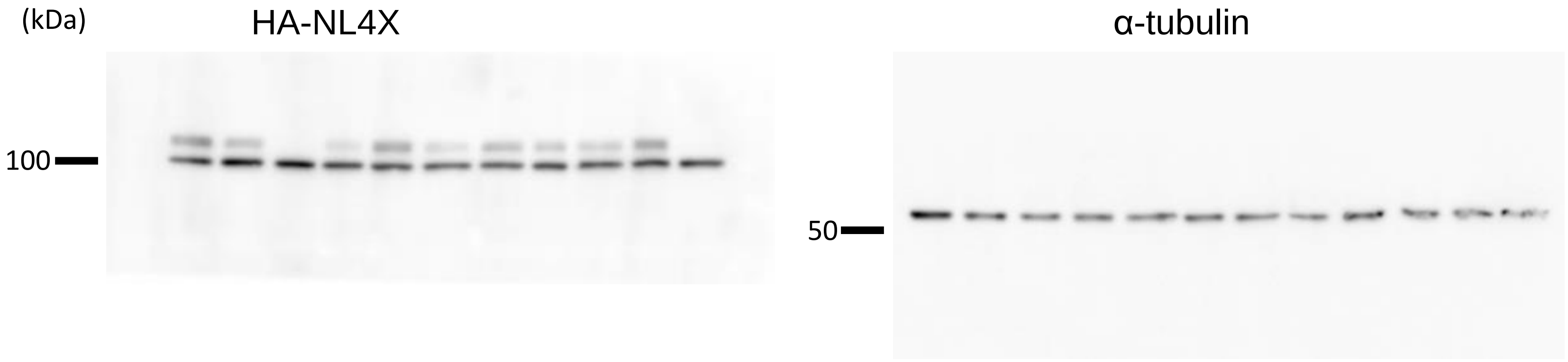


## Figure 4D

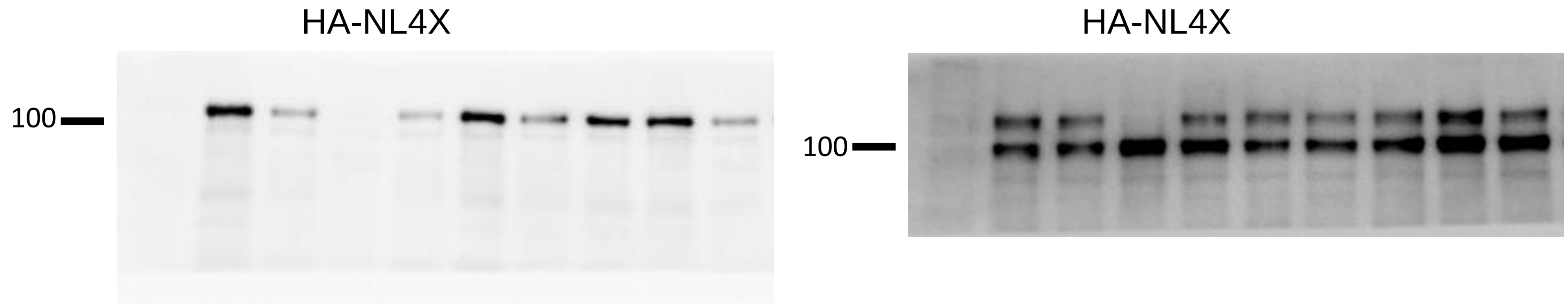

## Figure 4F

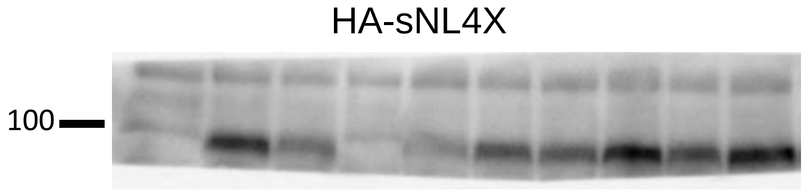


## Figure 5A

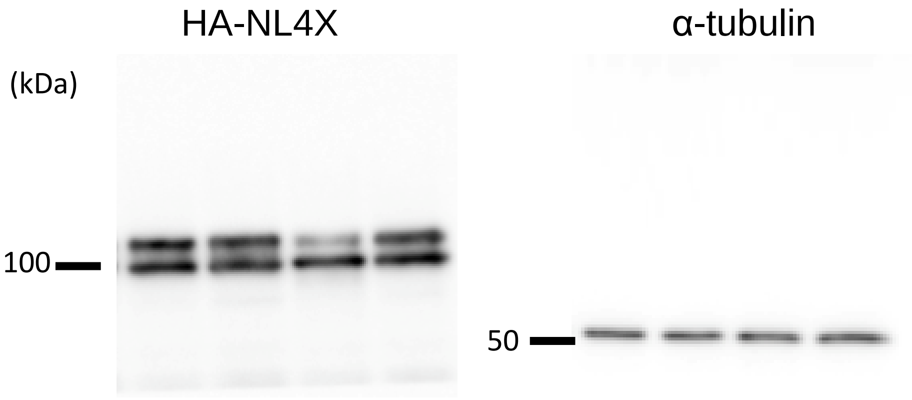


## Figure 5C

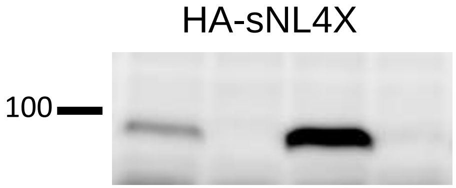


## Figure 5D

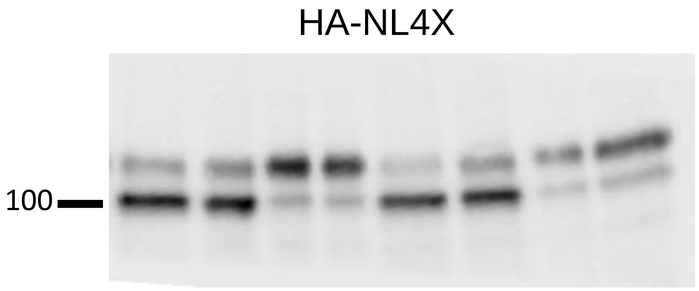

## Figure 6A

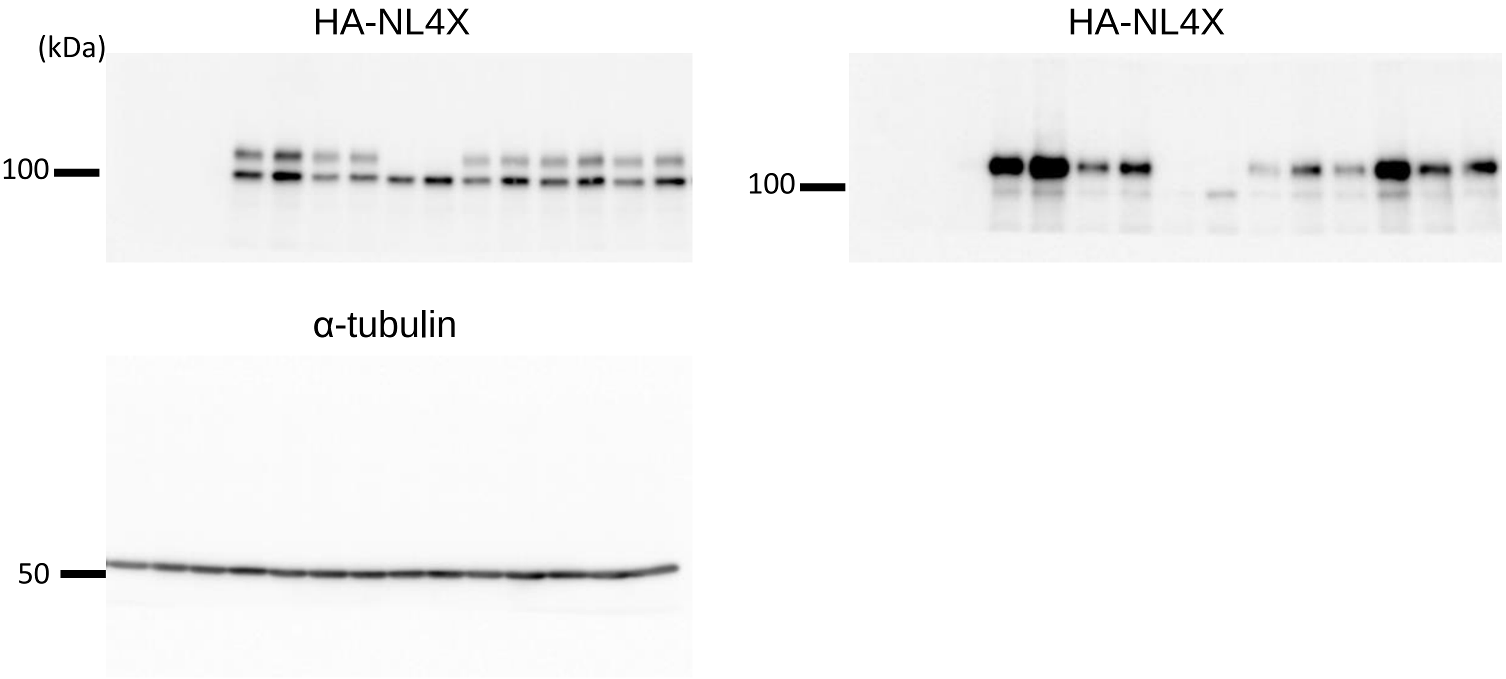


## Figure 6D

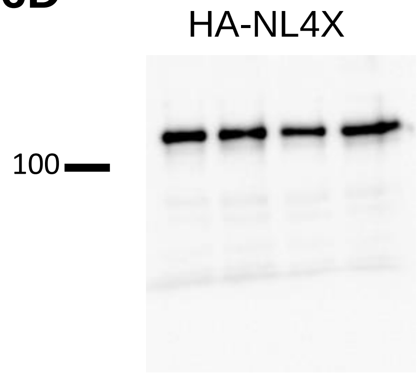

Supplement: Supplementary file 1 — Additional file 1:. Supplemental figures. [file 13229_2020_373_MOESM1_ESM.pdf]
